# Prognostic Significance of Ultralow (UL) Prostate-Specific Antigen (PSA) in Patients with Metastatic Hormone-Sensitive Prostate Cancer (mHSPC): A Single Institution and Tertiary Cancer Center Experience

**DOI:** 10.3390/medicina61122110

**Published:** 2025-11-27

**Authors:** Petar Suton, Višnja Gregov

**Affiliations:** Division of Oncology and Radiotherapy, University Hospital Dubrava, Avenue Gojko Šušak 6, 10000 Zagreb, Croatia

**Keywords:** prostate cancer, metastatic disease, prostate-specific antigen, survival

## Abstract

*Background and objectives:* Prostate-specific antigen (PSA) concentration is considered an important prognostic marker, with rapid and large reductions associated with a favorable outcome and prolonged survival. *Material and Methods:* We conducted a single institution and tertiary cancer center retrospective analysis of metastatic hormone-sensitive prostate cancer (mHSPC) patients undergoing active treatment at Division of Oncology and Radiotherapy, University Hospital Dubrava, between December 2022 and August 2025. This study aimed to assess the association between the PSA levels and survival in mHSPC patients undergoing active treatment. *Results:* A total of 42 (59.2%) in our cohort achieved UL PSA levels, while 29 patients (40.8%) had PSA levels > 0.2 ng/mL. Cox regression analysis identified age > 71 years at the diagnosis of mHSPC and UL PSA levels as statistically significant factors associated with favorable outcome. *Conclusions:* Our real-world data demonstrated that UL PSA is a favorable prognostic factor associated with prolonged survival and improved prognosis. However, we identified patients achieving UL PSA levels, who experienced radiographic progression. Our finding suggests that even among “best” PSA responders, some might develop resistant clones that manifest via imaging progression without PSA rise.

## 1. Introduction

The addition of androgen receptor-targeted agents (ARTA) to androgen deprivation therapy (ADT) has significantly improved the prognosis of metastatic hormone-sensitive prostate cancer (mHSPC) compared to ADT alone [1,2,3].

The prostate-specific antigen (PSA) concentration is considered an important prognostic marker, with rapid and large reductions associated with a favorable outcome and prolonged survival. A post hoc analysis of a randomized controlled trial (TITAN study) involving 1052 patients with mHSPC demonstrated that achieving a PSA decline to ≤0.2 ng/mL was associated with improved clinical outcomes [4]. Specifically, patients who reached a low PSA level had significantly longer overall survival (OS), radiographic progression-free survival (rPFS), time to PSA progression, and time to castration resistance, compared to those who did not achieve such a decline. Notably, a PSA level ≤ 0.02 ng/mL (UL2) was particularly predictive of favorable outcomes.

A real-world multi-center study involving 193 patients demonstrated that achieving UL PSA levels (<0.02 ng/mL) was associated with markedly improved outcomes [5]. Specifically, the 18-month overall survival (OS) rate was 100% for patients with UL PSA levels, compared to 94.4% for those with PSA levels between 0.02 and 0.2 ng/mL and 67.7% for those with PSA > 0.2 ng/mL. Similarly, the radiographic progression-free survival (rPFS) rate at 18 months was 100% in the UL PSA group, compared to 93.5% and 50.7% in the other two groups, respectively. The multivariate analysis confirmed that the UL PSA levels were independently associated with both the OS (hazard ratio [HR] 0.164, *p* = 0.0027) and rPFS (HR 0.089, *p* < 0.0001).

In a retrospective study of 129 patients with metastatic castration-resistant prostate cancer (mCRPC) treated with enzalutamide, achieving a PSA decline of ≥50% was associated with improved overall survival [6]. Specifically, patients receiving enzalutamide had a median OS of 7.8 months supporting the prognostic value of PSA declines in heavily pretreated patients.

We conducted a single institution and tertiary cancer center retrospective analysis of mHSPC patients undergoing active treatment between December 2022 and August 2025. This study aimed to assess the association between the PSA levels and survival in mHSPC patients undergoing active treatment with ADT plus ARTA or triplet therapy (with abiraterone or darolutamide). Other factors were analyzed in order to establish variables associated with the final outcome. Furthermore, additional effort was made to determine the factors associated with the UL PSA occurrence and to analyze cases with progression despite a biochemical response.

## 2. Materials and Methods

The study was approved by the Ethical Committee of the University Hospital Dubrava, Zagreb, Croatia. Follow-up intervals were calculated in months from the date of first treatment at our institution (gonadotropin-releasing hormone (GhRH) agonist therapy or bilateral orchiectomy) to the date of the last follow-up/death from any cause (OS) or signs of radiographic progression (rPFS). Patient monitoring was concluded on 1 August 2025. PSA levels were stratified by the following ranges: ultralow (UL) PSA (<0.2 ng/mL) and non-UL PSA > 0.2 ng/mL. Due to the relatively small numbers, no additional stratification between UL2 PSA (<0.02 ng/mL) and UL1 PSA (0.02–0.2 ng/mL) was conducted, and both ranges were included within the UL PSA group. The PSA levels were measured using the Abbott ARCHITECT^®^ Total PSA assay (Abbott Park, IL, USA) at the University Hospital Dubrava Clinical Laboratory. The assay has a detection limit of 0.01 ng/mL, allowing accurate quantification of very low PSA levels. The PSA was measured monthly in all patients, and the earliest time point achieving UL PSA (<0.2 ng/mL) during treatment was recorded for analysis. All samples were processed according to the manufacturer’s instructions and standard laboratory quality control procedures. All eligible patients were included consecutively during the study period to minimize the selection bias. There were no missing data for any variables. There were no strict criteria regarding radiographic assessment after treatment initiation; however, patients experiencing PSA decline received CT scans within one year from the start of the treatment. In cases of symptomatic disease and/or PSA rise, assessment was conducted within a six-week period. RECIST (Response Evaluation Criteria in Solid Tumors) criteria were used to define the radiographic progression. Treatment with ARTA started within one month from ADT initiation/bilateral orchiectomy in all patients. In the triplet therapy group, all patients received androgen-deprivation therapy (LHRH agonist or underwent orchiectomy) before the start of systemic therapy: patients received six cycles of docetaxel (75 mg per square meter of body-surface area on day 1 and every 21 days), with prednisone or prednisolone. The recommended premedication to prevent docetaxel-related hypersensitivity reactions and fluid retention was oral dexamethasone, administered at a dose of 8 mg at 12 h and 3 h before infusion. For patients receiving LHRH agonists, the use of these agonists in combination with a first-generation antiandrogen for at least 3 weeks before was performed. First-generation antiandrogen therapy was discontinued on the day of LHRH agonist initiation. Darolutamide/abiraterone usually started on day 1 of the systemic therapy schedule. The chi- square test was used to assess the relationship of PSA status (UL PSA vs. non-UL PSA) with other clinical variables stratified at median or in their respective categories. The logistic regression was used to assess the independent predictors of UL PSA status. Survival outcomes were estimated using the Kaplan–Meier method and compared with the log-rank test. Associations between baseline variables and outcomes were evaluated using Cox proportional hazards regression. Proportional hazard assumptions were assessed using Schoenfeld residuals, and potential multi-collinearity among predictors was checked by examining the correlation matrices. The number of covariates included in the final model was limited to the following variables: age, UL PSA, metastatic timing, and disease volume. This adjustment was conducted in order to improve the stability of the hazard ratio estimates and to reduce the risk of overfitting. All tests were two-sided, and *p*-values < 0.05 were considered statistically significant. All analyses were performed using MedCalc version 23.2.1 (MedCalc Software Ltd., version 23.4 © 2025, Ostend, Belgium).

## 3. Results

**Figure 1:**
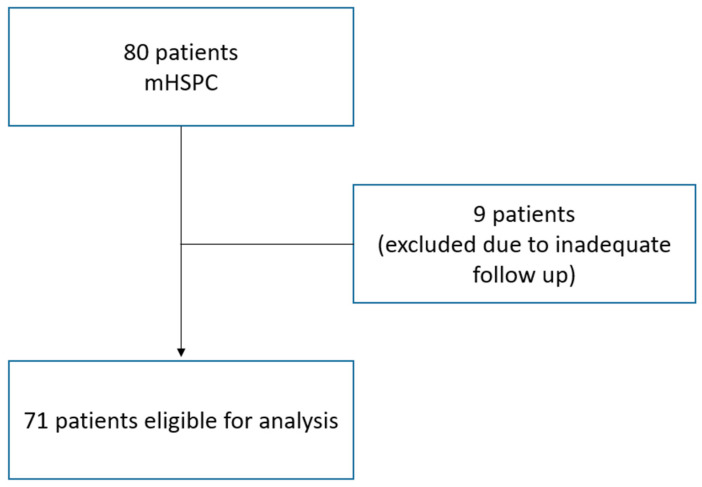
Flowchart of the included patients.

In a 32-month period, 80 patients with mHSPC were treated at the Division of Oncology and Radiotherapy University Hospital, Dubrava. After excluding patients with inadequate follow-up periods (<6 months, *n* = 9) 71 patients were available for further analysis (Figure 1).

The baseline characteristics of the patients are summarized in Table 1.

A total of 42 (59.2%) patients in our cohort achieved UL PSA levels, while 29 patients (40.8%) had PSA levels > 0.2 ng/mL. In the UL PSA cohort, 33 (78.6%) patients achieved PSA levels < 0.2 ng/mL within 6 months from ARTA introduction, while seven (16.7%) patients had their first UL PSA levels detected from 6 to 12 months from treatment initiation, and only two (4.7%) patients had a UL PSA starting from the 12th month of treatment. The median age was 71 years (range 49–94 years), and most of the patients presented with de novo metastatic disease (67.6%, *n* = 48). Enzalutamide was the most common ARTA treatment used in our patients (*n* = 46, 64.8%), followed by triplet (*n* = 11, 15.5%), apalutamide (*n* = 7, 9.8%) and abiraterone (*n* = 7, 9.8%). A total of 64.8% of our cohort of patients had a Gleason score 8 or more. Gonadotropin-releasing hormone (GnRh) was the most common basic treatment in order to achieve androgen deprivation (60.6%, *n* = 43), while 28 (39.4%) patients underwent bilateral orchiectomy.

**Figure 2:**
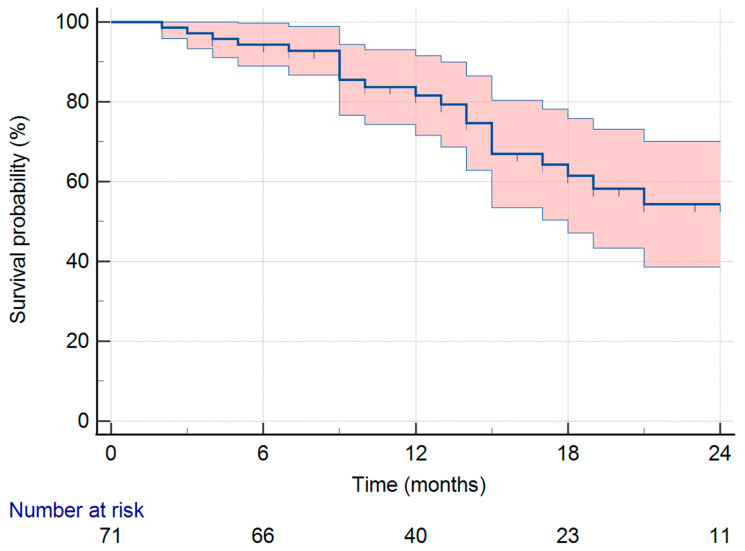
Survival rate of the cohort with 95% CI.

The 18-month survival rate was 61.5% (Figure 2).

**Figure 3:**
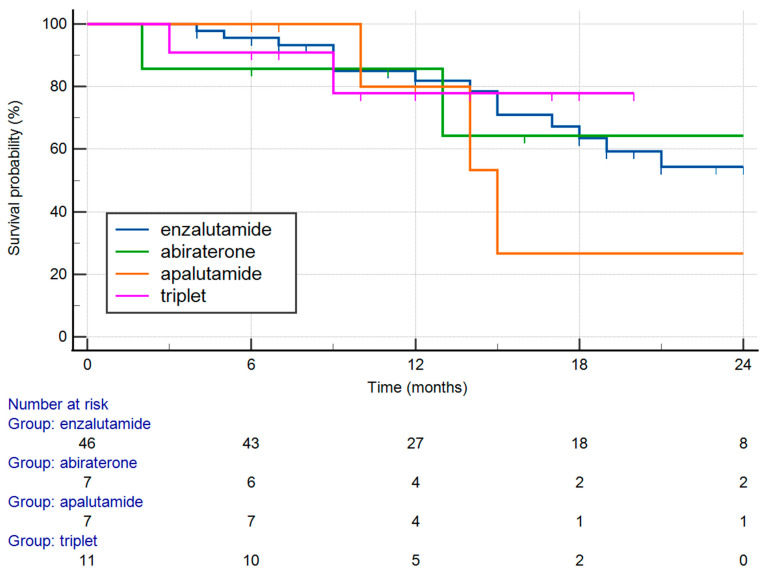
Survival by treatment type.

The survival by treatment type is shown in Figure 3. There was no significant difference in the final outcome with respect to the treatment modality (*p* = 0.845).

Cox regression analysis identified age > 71 years at the diagnosis of mHSPC and UL PSA levels as a statistically significant factor associated with a favorable outcome (Table 2.

**Figure 4:**
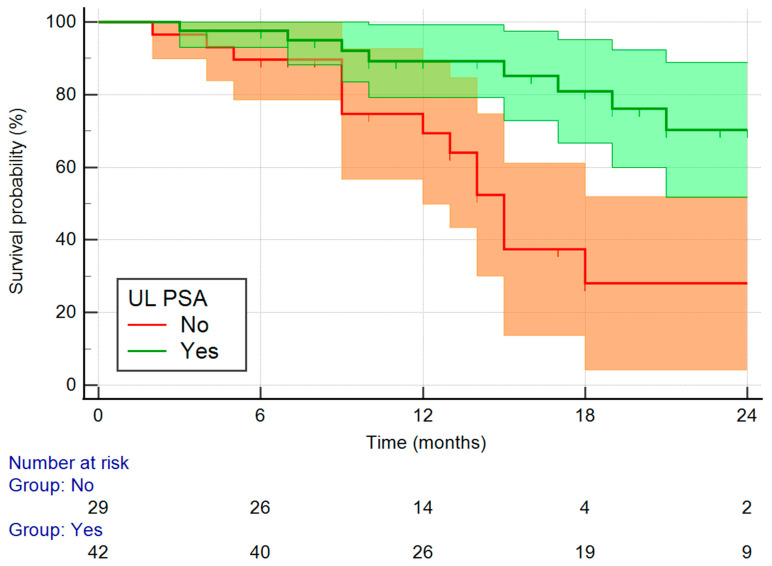
Survival rate with respect to UL PSA status with corresponding 95% Cis.

The Kaplan–Meier survival curve of patients with respect to the UL PSA is shown in Figure 4. In the UL PSA cohort, the median survival was not reached, while the non-UL PSA patients had a median survival of 15 months (95% CI 12–18 months).

Factors associated with UL PSA levels in the univariate model were age < 71, a metachronous pattern of metastatic disease, and a low-volume metastatic disease (Table 3). In the logistic regression model, the only independent predictor of UL PSA status was age < 71 years (Table 4).

Patients were followed up from 6 to 32 months (median 13 months). At the end of the follow-up period, 22 (31%) patients died from disease/other causes. Interestingly, in our cohort, 5 out of 71 patients (7% of the cohort, 11.9% of UL PSA group) achieving UL PSA levels experienced radiographic progression and died from disease with a median OS of 13.5 months. In this subgroup, all patients had high risk/volume symptomatic disease with rapid clinical deterioration. The characteristics of these patients are summarized in Table 5.

## 4. Discussion

Introduction of ARTA in the treatment of prostate cancer has significantly changed the outcomes of patients in a metastatic setting [1,2,3]. However, despite all therapeutic advances, when the disease reaches the castration-resistant stage, there is limited efficacy in the existing treatment options. Furthermore, there are no identified biomarkers that can tailor the treatment of these patients. In addition, the choice of the optimal treatment is determined by numerous factors such as the disease volume and risk (CHAARTED/LATITUDE criteria, disease dynamics (metastatic disease from previously treated locoregional disease vs. de novo mHSPC), and the patient’s features (ECOG PS, comorbidities, age at diagnosis, etc.) [3,7,8]. However, there are no general recommendations with respect to the optimal treatment in terms of the previously mentioned characteristics.

Recently, with the development of ultrasensitive assays, it has become increasingly common to detect PSA levels below 0.2 ng/mL.

Our results confirm that achieving an UL PSA level in mHSPC is associated with favorable outcomes (Table 2, HR 0.16, *p* = 0.0005 in alignment with prior reports [4,5,9,10]. In addition, we have identified a similar incidence of UL PSA to that in previously mentioned cohorts.

This study found that a younger age (<71 years) predicted UL PSA (Table 4), whereas older age (>71 years) was associated with improved OS (Table 2). Interestingly, all patients who achieved UL PSA but subsequently developed radiographic progression were younger than 71 years of age (Table 5). This pattern suggests that while younger patients may respond biochemically, their disease might be biologically more aggressive, leading to earlier progression despite initial PSA suppression. In contrast, older patients may have a less aggressive disease biology or different treatment patterns that translate into longer OS. These findings highlight potential confounding or effect modification by disease burden and treatment selection, emphasizing the need for larger prospective studies to disentangle the effects of age, disease characteristics, and treatment response on the long-term outcomes.

As mentioned, we identified a subgroup of patients experiencing radiographic progression despite UL PSA levels (Table 5). This phenomenon—radiographic progression in the absence of PSA progression—has been described in various prostate cancer settings [11,12,13]. In our cohort, all these patients were characterized by high-risk disease features and rapid clinical deterioration, suggesting that, despite achieving profound biochemical responses, aggressive tumor biology can drive disease progression. This observation raises important questions about the prognostic reliability of the PSA level as a surrogate for disease control and suggests heterogeneity in disease biology and patterns of cancer resistance.

What is less clear from the literature is whether this discordance is characteristic of patients who achieve UL PSA levels. Most of the published analyses examine discordance across all PSA strata, not specifically in a UL PSA cohort [14,15]. Our finding suggests that even among the “best” PSA responders, some might develop resistant clones that manifest via imaging progression without a PSA rise. To our knowledge, this is one of the first comprehensive reports in the literature describing and analyzing radiographic progression and subsequent mortality in patients who achieved UL PSA levels for metastatic prostate cancer.

Furthermore, these findings underscore the limitations of using the PSA response, even at UL levels, as the sole marker for treatment efficacy and disease monitoring. The following clinical considerations are recommended:

- Radiographic surveillance remains essential even in UL PSA achievers, as a subset may develop non-biochemical progression.
- Risk stratification tools incorporating imaging, baseline clinical characteristics (e.g., high-volume/high risk disease), and genomic data may help identify at-risk patients.
- Molecular profiling (e.g., cfDNA, RNA expression) in progressing patients may uncover mechanisms of resistance, including neuroendocrine (de)differentiation or AR-independent pathways [8,10].
- Alternative endpoints: trials and clinical practice should use other endpoints that include imaging and symptomatic clinical progression, not just PSA dynamic.

In terms of outcomes, the 18-month OS rate of 61.5% appears lower than expected for modern mHSPC cohorts. Compared with pivotal RCTs in metastatic prostate cancer such as CHAARTED, LATITUDE, STAMPEDE, and ENZAMET [3,7,8,16]—our cohort represents a higher-risk population, with approximately 79% of patients presenting with high-volume disease. These trials typically demonstrated 18-month OS rates exceeding 80–90%. This discrepancy likely reflects differences in patient selection and real-world practice, including higher disease burden, potential delays in treatment initiation, and heterogeneity in systemic therapy intensity. Additionally, the limited follow-up duration in our cohort may have contributed to an apparent underestimation of the survival outcomes. These findings underscore the challenges of replicating RCT outcomes in unselected, high-risk, and real-world populations and highlight the need for strategies to optimize treatment in this group.

In addition, in this study, the treatment type did not significantly affect the OS (Figure 3, *p* = 0.845). However, this finding should be interpreted with caution, due to the limited sample size and potential selection bias. The small cohort may have reduced the statistical power to detect meaningful differences between treatment groups. Additionally, the non-randomized design could have introduced confounding factors influencing the treatment selection and patient outcomes. Therefore, while the results suggest no clear survival advantage among the treatment modalities assessed, further studies with larger well-balanced cohorts are needed to validate these observations and better define the impact of treatment type on survival outcomes.

Further, we have also evaluated potential predictors of achieving UL PSA levels. As previously mentioned, the multivariate analysis identified age < 71 years as the only independent predictor of achieving a UL PSA response. This finding is partially divergent from previously published studies, where tumor-related factors such as the baseline PSA levels and timing of metastatic presentation were more commonly associated with deeper PSA responses.

In a recent multicenter cohort study by Martínez-Corral et al., which included 586 mHSPC patients treated with ADT and ARTA, the authors specifically examined predictors of achieving deep PSA responses, including a PSA nadir < 0.02 ng/mL at 6 months (TITAN criteria) and <0.2 ng/mL (SWOG criteria) [17]. They found that a baseline PSA < 50 ng/mL and metachronous metastatic disease were significantly associated with achieving UL PSA levels in multivariate analysis. In contrast, age was not reported as a significant predictor in their model, although it was included as a covariate. These differences may suggest cohort-specific variations in patient and disease characteristics or differing statistical power between studies.

Similarly, López-Abad et al. reported that low-volume disease, metachronous metastases, and M1a staging were associated with a higher likelihood of achieving UL PSA in a multicenter real-world study of 193 mHSPC patients treated with apalutamide plus ADT [5]. Again, age was not found to be a significant factor. These findings reinforce the consistent role of disease burden and metastatic timing in predicting depth of PSA response.

Furthermore, Sweeney et al. demonstrated that the addition of enzalutamide for all patients with metachronous metastases and low-volume disease was associated with higher survival rates [18]. The study did not identify age as a significant independent predictor. Collectively, these findings underscore the importance of disease biology, particularly the timing and extent of metastatic spread, in influencing treatment response.

Our identification of younger age (<71 years) as the only independent predictor of UL PSA contrasts with the aforementioned studies and may reflect differences in patient demographics, treatment regimens, or sample size. One potential explanation is that younger patients may exhibit better treatment adherence, more robust immune responses, or even more favorable tumor biology that facilitates deeper PSA declines. Alternatively, our study may have lacked sufficient variability in disease characteristics such as baseline PSA or metastatic volume, limiting our ability to detect associations observed in larger cohorts. However, while younger patients were more likely to achieve UL PSA in our study, age < 71 years was negative prognostic factor with respect to survival in multivariate model. One of the potential explanations for such phenomenon might be that all patients achieving UL PSA and experiencing radiographic progression were within this age group.

### Limitations of the Study

This study has all of the weaknesses associated with a retrospective design of a study. In this retrospective series, we pooled UL2 (<0.02 ng/mL) and UL1 (0.02–0.2 ng/mL) groups to define UL PSA < 0.2. Since the prior literature has shown <0.02 ng/mL to be especially prognostic [4,5], this pooling may mask clinically meaningful differences. While several pivotal trials in mHSPC, such as TITAN and ARCHES [2,19], have demonstrated the prognostic value of early PSA decline, the optimal threshold for defining deep biochemical response remains unknown. Many studies have used ≤0.2 ng/mL as an indicator of favorable response (i.e., SWOG cutoffs), whereas others have adopted more stringent definitions (e.g., ≤0.02 ng/mL) [4,5]. In our cohort, the <0.2 ng/mL cutoff was selected primarily due to the small sample size, which limited the feasibility of using narrower thresholds while maintaining meaningful subgroup comparisons. This definition also aligns with previously reported cutoffs for deep PSA suppression in mHSPC, facilitating comparison with established studies while ensuring adequate statistical power.

Additionally, the absence of standardized imaging criteria represents an important limitation of this study. CT scans were performed annually for patients with stable or undetectable PSA levels, but earlier imaging (within six weeks) was performed in cases of symptomatic progression or PSA rise. This approach introduces potential verification (assessment) bias in the evaluation of radiographic progression-free survival (rPFS), as the timing of imaging—and thus the likelihood of detecting progression—was dependent on the PSA dynamics. To improve the interpretability, future studies should implement predefined imaging schedules independent of PSA dynamic or perform sensitivity analyses, restricted to patients with uniform imaging intervals to minimize this bias. Further, although imaging intervals were non-uniform due to the real-world practice patterns of the study, the use of standardized RECIST criteria ensured consistency and reproducibility across all assessments. We believe that this approach strengthens the validity of our rPFS analyses. Additionally, ARTA was initiated within one month after ADT or orchiectomy, which, if modeled as a fixed exposure, may introduce immortal time bias. Although immortal time bias can occur when treatment initiation is delayed or modeled as a fixed exposure, the results of this study substantially reduce that risk. In our cohort, patients received triplet therapy-initiated androgen deprivation therapy (ADT), docetaxel, and the androgen receptor–targeted agent (ARTA) in close temporal sequence, typically within the same treatment cycle. This synchronous initiation minimizes any “immortal” period, during which patients would have needed to survive before being classified as treated. As a result, the treatment exposure was effectively determined at baseline rather than being contingent on post-baseline survival. Consequently, the likelihood of immortal time bias influencing survival estimates is low. Nonetheless, acknowledging this potential remains important, and future analyses using time-dependent modeling could further confirm the robustness of these findings. Furthermore, an important limitation of this study is the relatively small number of outcome events, with only 22 deaths observed during the follow-up period. Although a multivariable Cox proportional hazards model was applied, the inclusion of several covariates resulted in fewer than 10 events per variable. This limited number of events may compromise the stability and precision of the estimated hazard ratios, increasing the likelihood of model overfitting. As a result, the reported associations should be interpreted with caution, and further studies with larger event numbers are needed to confirm these findings.

In addition, the relatively small number of patients precludes a clear conclusion, and the data and results need to be validated within controlled and well-balanced trials. However, this is single institution and tertiary cancer center study with strict follow-up, and we believe that these results contribute to the existing body of knowledge in the context of confirming the prognostic significance of UL PSA, as well as analysis of other clinical and histopathological factors.

Furthermore, we believe that the identification of a subgroup of UL PSA patients with radiographic progression represents a novel and clinically relevant nuance. It underscores that PSA suppression, even when pronounced and rapid, does not universally guarantee durable disease control. These patients may represent a biologically distinct therapy-resistant subset. Therefore, prudent imaging surveillance and consideration of escalation strategies are warranted, and future translational and prospective studies should focus on understanding and managing this subgroup of patients. Despite achieving UL PSA < 0.2 ng/mL, a subset of patients in our cohort experienced radiographic progression, highlighting a potential discrepance between PSA response and disease biology. One plausible explanation is the development of neuroendocrine differentiation or AR-independent tumor clones, which may contribute to tumor growth and progression despite androgen deprivation and profound PSA responses. Additionally, PSA primarily reflects the activity of luminal androgen-sensitive tumor cells and may fail to capture tumor heterogeneity or aggressive cancer variants, including small-cell or ductal differentiation. These observations underscore the limitations of PSA as a sole biomarker and suggest that radiographic monitoring remains critical, particularly in high-risk or heavily pre-treated patients. Future studies integrating molecular profiling and circulating tumor biomarkers may help identify patients at risk for progression in the subset of patients experiencing UL PSA.

## 5. Conclusions

In summary, our real-world data demonstrated that UL PSA is a favorable prognostic factor associated with prolonged survival and improved prognosis. However, radiographic progression can occur in a small, but clinically important, subset of patients who achieve UL PSA levels. This challenges the prevailing assumption that deep PSA suppression is a universally positive prognostic marker and emphasizes the continued importance of imaging surveillance and comprehensive risk stratification in the management of metastatic prostate cancer.

## Figures and Tables

**Table 1:** Patients characteristics.

Characteristics	
Median age (range), years	71 (49–94)
Median iPSA (range), ng/mL	87 (1.5–6903)
Gleason score (GS)	
<7	25 (35.2%)
≥8	46 (64.8%)
Disease volume	
Low	15 (21.1%)
High	56 (78.9%)
Metastases timing	
Synchronous	48 (67.6%)
Metachronous	23 (32.4%)
UL PSA	42 (59.2%)

**Table 2:** Multivariate predictors of survival.

Covariate	b	SE	Wald	*p*	Exp(b)	95% CI of Exp(b)
Years (median)	−1.0504	0.4813	4.7639	**0.0291**	0.3498	0.1362 to 0.8984
Metastases timing (synchronous vs. metachronous	−0.2732	0.5471	0.2494	0.6175	0.7609	0.2604 to 2.2237
Disease volume (low vs. high)	−0.7077	0.6844	1.0691	0.3011	0.4928	0.1288 to 1.8848
UL PSA	−1.8156	0.5235	12.0297	**0.0005**	0.1627	0.0583 to 0.4540

**Table 3:** Factors associated with UL PSA levels in univariate analysis.

Variable	Non-UL PSA	UL PSA	*p* Value
Years			**0.0190**
<71	9	25	
>71	20	17	
GS			0.1070
<7	7	18	
≥8	22	24	
PSA median			0.318
<87	8	26	
>87	21	16	
Metastatic pattern			**0.0244**
Metachronous	5	18	
Synchronous	24	24	
Disease volume			**0.0154**
Low	2	13	
High	27	29	
Type of therapy			0.715
Enzalutamide	18	28	
Abiraterone	4	3	
Apalutamide	2	5	
Triplet	5	6	

**Table 4:** The logistic regression-independent predictors of UL PSA.

Covariate	Coefficient	SE	Wald	*p*
Years (median)	−1.88853	0.68060	7.6995	**0.0055**
Gleason score (<7 vs. ≤8)	−0.28438	0.63290	0.2019	0.6532
iPSA_(median)	−1.22873	0.81816	2.2555	0.1331
GhRh vs. bilateral orchiectomy	0.19384	0.63967	0.09183	0.7619
Metastases timing (synchronous vs. metachronous	−0.59124	0.85286	0.4806	0.4882
Disease volume (low vs. high) ^a^	−0.89503	0.92733	0.9316	0.3345
Treatment type	−0.12416	0.26305	0.2228	0.6369
Constant	5.12870	2.32747	4.8556	0.0276

^a^ per CHAARTED criteria. CHAARTED defines high-volume disease by the presence of visceral metastases or four or more bone lesions, while LATITUDE defines high-risk disease by meeting at least two of three factors: Gleason score of 8 or higher, three or more bone lesions, and visceral metastases.

**Table 5:** Patients with UL PSA and radiographic progression.

Years	De Novo mHSPC	Gleason Score	Grade Group	iPSA	Treatment	Lowest PSA	Survival (Months)
56	Yes	4 + 4	4	114	apalutamide	0	15
68	Yes	4 + 4	4	86.54	enzalutamide	0.18	22
71	No	4 + 4	4	4.66	enzalutamide	<0.01	17
68	No	3 + 5	4	27	apalutamide	<0.01	10
60	Yes	4 + 4	4	709.8	enzalutamide	0.2	9

## Data Availability

The data are not publicly available due to ethical reasons. Further inquiries should be directed to the corresponding author.

## Abbreviations

The following abbreviations are used in this manuscript:

PSA	prostate-specific antigen
mHSPC	metastatic hormone-sensitive prostate cancer
mCRPC	metastatic castration-resistant prostate cancer
ARTA	androgen receptor-targeted agents
ADT	androgen deprivation therapy
GnRH	gonadotropin-releasing hormone
OS	overall survival
rPFS	radiographic progression-free survival
cfDNA	cell free deoxyribonucleic acid
RNA	ribonucleic acid
